# P05-04 Physical activity level and sedentary time prior to cardiac ward admission among patients with cardiovascular disease and its association to all-cause mortality

**DOI:** 10.1093/eurpub/ckac095.071

**Published:** 2022-08-29

**Authors:** Amanda Ek, Lena Kallings, Mattias Ekström, Mats Börjesson, Örjan Ekblom

**Affiliations:** 1 Swedish school of sport and Health science, Stockholm, Sweden; 2 Åstrand Laboratory of Work Physiology, Swedish School of sport and Health sciences, GIH, Stockholm, Sweden; 3 Department of Clinical Sciences, Danderyd Hospital, Division of Cardiovascular Medicine, Stockholm, Sweden; 4 Institute of Medicine, Sahlgrenska academy, University of Gothenburg, Gothenburg, Sweden

**Keywords:** physical exercise, sedentary behaviour, heart diseases, survival

## Abstract

**Background:**

Low physical activity (PA) level and high sedentary time (SED) have been associated to cardiovascular (CVD) morbidity and mortality. Routinely assessing the PA-level of patients being admitted to hospital has been proposed. The aim was to explore PA-level and SED among patients prior to cardiac ward admission and whether this can predict all-cause mortality.

**Methods:**

A longitudinal observational study of patients with ischemic heart disease, heart failure, cardiac arrhythmia, valvular heart disorder and inflammatory heart diseases treated on cardiac wards (2015-2016) in Stockholm, Sweden. Data on PA-levels and SED prior to admission were collected by validated questionnaires during inpatient care. PA level a regular week was calculated by an index (3-19 points) including everyday PA and exercise. The cut-off of insufficiently physically active was set to > 9 points. Individuals' reporting ≥7 hours of sitting a normal day were categorised as high SED. Differences in PA-level and SED between different diagnose groups were explored by Benjamini-Hochberg procedure. The associations between PA-level and SED with all-cause mortality were analysed using cox regressions, adjusting for age, sex, diagnosis group, education level, disposable income, smoking status, alcohol consumption and eating habits.

**Results:**

Among 1148 patients with CVD, approximately 56% were considered as insufficiently physically active (>9 points). In addition, approximately half the study population were categorized as high SED (≥7 hours per day). There were differences in PA-level and SED between the various cardiovascular diagnoses, with individuals with heart failure and valvular heart disorder being in general more inactive and having higher levels of SED. A total of 200 deaths occurred during a median follow-up time of 2.6 years. The mortality was higher among those categorised as insufficiently physically active (HR 1.49, 95% CI 1.08-2.07) or high SED (HR 1.79, 95% CI 1.32-2.43) compared to those reporting sufficient PA and low SED, respectively.

**Conclusion:**

A high amount reported insufficient PA and a high amount of SED preceding hospitalisation. There was an association between PA (negatively) and SED (positively) with all-cause mortality among patients with CVD. This highlights the prognostic value of assessing patients' PA-level and SED in clinical practice.

